# Reported reasons for not using a mosquito net when one is available: a review of the published literature

**DOI:** 10.1186/1475-2875-10-83

**Published:** 2011-04-11

**Authors:** Justin Pulford, Manuel W Hetzel, Miranda Bryant, Peter M Siba, Ivo Mueller

**Affiliations:** 1 Papua New Guinea Institute of Medical Research (PNGIMR), PO Box 60, Goroka, EHP 441, Papua New Guinea; 2 School of Population Health, University of Queensland, Brisbane, Australia; 3 Population Services International (PSI), Papua New Guinea; 4 Barcelona Centre for International Health Research, Barcelona, Spain

## Abstract

**Background:**

A review of the barriers to mosquito net use in malaria-endemic countries has yet to be presented in the published literature despite considerable research interest in this area. This paper partly addresses this gap by reviewing one component of the evidence base; namely, published research pertaining to self-reported reasons for not using a mosquito net among net 'owning' individuals. It was anticipated that the review findings would potentially inform an intervention or range of interventions best suited to promoting greater net use amongst this group.

**Method:**

Studies were sought via a search of the Medline database. The key inclusion criteria were: that study participants could be identified as owning a mosquito net or having a mosquito net available for use; that these participants on one or more occasions were identified or self-reported as not using the mosquito net; and that reasons for not using the mosquito net were reported. Studies meeting these criteria were included irrespective of mosquito net type.

**Results:**

A total of 22 studies met the inclusion criteria. Discomfort, primarily due to heat, and perceived (low) mosquito density were the most widely identified reason for non-use. Social factors, such as sleeping elsewhere, or not sleeping at all, were also reported across studies as were technical factors related to mosquito net use (i.e. not being able to hang a mosquito net or finding it inconvenient to hang) and the temporary unavailability of a normally available mosquito net (primarily due to someone else using it). However, confidence in the reported findings was substantially undermined by a range of methodological limitations and a dearth of dedicated research investigation.

**Conclusions:**

The findings of this review should be considered highly tentative until such time as greater quantities of dedicated, well-designed and reported studies are available in the published literature. The current evidence-base is not sufficient in scope or quality to reliably inform mosquito net promoting interventions or campaigns targeted at individuals who own, but do not (reliably) use, mosquito nets.

## Background

Mosquito nets, most commonly in the form of insecticide-treated mosquito nets (ITN), are a central component of current global malaria control initiatives. The evidence in support of ITNs as a malaria control strategy is overwhelming. Systematic reviews of randomized controlled trials confirm a significant reduction in individual risk of malaria-related morbidity and mortality associated with ITN use [1,2]. Individuals' not sleeping under an ITN, but living within an area with high ITN coverage, have also been shown to be at decreased risk of infection due to the resulting reduction in overall malaria transmission [3,4]. Encouragingly then, and largely as a result of donor-funded distribution programmes, ITN ownership has substantially increased in many malaria-endemic countries over the past decade [5-8].

Mosquito net ownership is far from universal despite the aforementioned gains. Ownership rates remain low in many malarious regions or amongst particular groups in malarious regions [9-12]. Furthermore, mosquito net ownership in itself is not synonymous with utilization. For example, in one study in Niger, West Africa, as few as 33% of available mosquito nets in mosquito net owning households were used the night prior to survey [13]. The rate of mosquito net use in these ITN owning households was substantially less than 100% indicating factors other than availability were responsible for reported instances of mosquito net non-use. Other studies, again in contexts where fewer than 100% of household members reported mosquito net use, have reported between 15-50% of available nets going unused [14-17]. Thus, ownership is not the only obstacle to achieving the reductions in malaria morbidity and mortality associated with ITN use; rather, individuals who own (or who have available) mosquito nets must use them in order for the potential health impact to be fully realized.

Determining whether sub-optimal mosquito net utilization in a given population is due to a lack of availability or a failure to utilize available nets is operationally important in a malaria control context as it would inform the subsequent response [11]. This 'targeted' approach to mosquito net promotion is encapsulated in a recently proposed framework designed to inform "...evidence-based and country-specific strategies to increase population coverage with ITNs and work towards the interruption of malaria transmission" [18]. Three categories of mosquito net non-use are recognized within this framework: 1) living in households with no mosquito nets present; 2) living in households owning, but not hanging a mosquito net; and 3) living in households that have a mosquito net hanging but who are not sleeping under a mosquito net. Depending on which category is found to account for most or significant mosquito net non-use, the authors argue resulting interventions should variously focus on improving mosquito net availability (category 1), encouraging the hanging of mosquito nets (category 2), or targeting individuals to encourage use of an existing mosquito net (category 3) [18].

This framework usefully highlights the variability in reasons for mosquito net non-use and the importance of tailoring intervention strategies accordingly; however, the necessary interventions to promote greater mosquito net use among mosquito net 'owners' (category 2 and 3) are likely to be considerably more complex than the 'information, education or behaviour change communication' campaigns subsequently suggested [18]. For example, mosquito net non-use among mosquito net owning individuals has been variously attributed to practical barriers associated with erecting a mosquito net [19], the temporary unavailability of a normally available mosquito net [20] or a range of social factors that render mosquito net use impractical in the short-term [21]. An educational- or behaviour change-based intervention designed to promote greater mosquito net use is unlikely to be effective in these cases (in isolation, at least); rather, design modifications at the manufacturer level, the provision of additional mosquito nets or the promotion of complementary malaria control interventions may be better considered.

A review of the barriers to mosquito net use has yet to be presented in the published literature despite considerable research interest in this area. The aim of this paper, therefore, was to partly address this gap by reviewing one component of the evidence base; namely, published research pertaining to self-reported reasons for not using a mosquito net among individuals who have one available. It was anticipated that a focused review of this nature would highlight the current state of the literature pertaining to a specific population of mosquito net non-users and, pending the quality and scope of the available evidence, potentially inform an intervention or range of interventions best suited to promoting greater net use amongst this group.

## Method

### Search strategy

Studies were sought via a search of the Medline database. The reference period for the search was January 1990 to September 2010. The search was limited to English language publications and was conducted using the following keywords: mosquito net, bed net, ITN, LLIN and barrier, non use, reason, sleep, attitude, knowledge, practice, misuse, obstacle, perception, acceptability, reaction, belief. Further studies were also sought via a manual search of references listed in retrieved articles.

### Study selection

Studies were included in this review if they presented reasons for not using a mosquito net from an individual or individuals who were reported to own/have available a mosquito net. The key inclusion criteria were, therefore, that study participants could be identified as owning a mosquito net or having a mosquito net available to use, that these participants on one or more occasions were identified or self reported as not using the mosquito net and that reasons for not using the mosquito net were reported. Studies meeting these criteria were included irrespective of mosquito net type. Studies were excluded from review if the reported reasons for not using a mosquito net could not be attributed to a known mosquito net owner or if the same data were reported in another (included) publication. For convenience, individuals' who own a mosquito net or who have a mosquito net available for use are referred to as mosquito net 'owners' in the following review.

## Results

Publications presenting relevant data obtained from two broad types of study design were identified by the search criteria: namely, publications presenting data obtained in response to a structured survey question and publications presenting data obtained in response to some form of qualitative enquiry. The respective survey- and qualitative- data are reviewed separately below.

### Survey data

A total of 17 studies meeting the inclusion/exclusion criteria were identified in which reasons for not using a mosquito net, as reported by identified mosquito net owners (or the caregivers/household heads on behalf of mosquito net owners) in response to a structured survey question(s), were described. In eight of these 17 studies the proportion of respondents reporting each of the respective reasons for not using a mosquito net was not identified or the proportion was identified, but no denominator was provided or could be calculated [13,19,22-27]. These studies (non-specific) are reviewed separately from the remaining nine studies in which detailed 'reasons for not using a mosquito net' data were presented (specific).

The nine 'specific' studies are listed in Table 1 along with the respective study population, sample size, net type, non-use measure, the reported reasons for non-use, and the reported number of participants endorsing each of the listed reasons. For better comparability each reported reason was assigned to one of six 'reason for non-use' categories based in part on those reported in Alaii *et al *[20]. The assigned categories - availability (non-use due to the unavailability of a normally available net), discomfort (non-use due to personal discomfort), perceived (low) mosquito density (non-use due to a perceived low mosquito density), social (non-use due to factors associated with the individuals social environment), technical (non-use due to technical issues associated with hanging or using a net), and other (reasons for non-use that do not fall into the aforementioned categories) - are also presented in Table 1.

**Table 1:** Reported reasons for non-use as given by participants who own/have available a mosquito net

Study	Study Population	SS	Net Type	Non-use Measure	Reported Reasons for Non-use	N	Non-use Category
[20]^a^	Children < 5 yrs^b^	69^c^	ITN	Undefined	Physical barrier to net use^d^	23	Technical
					Heat	22	Discomfort
					Disruption of sleeping arrangements	16	Social
					Perceived mosquito density (PMD)	11	PMD
					Net temporarily unavailable^e^	9	Availability
					Caregiver not present^f^	8	Social
					Inconvenience^g^	4	Technical
					Forgetfulness	4	Other
					Cannot use since child is sick	3	Other
					Funeral affected net use	2	Social
					Slept elsewhere	1	Social
					Net is damaged	1	Other
					Net is in storage	1	Other
					Child fears ants will climb up net	1	Other
[21]^h^	Household members	294^i^	ITN	No use - previous night or week	Away on business or visiting relatives	44^j^	Social
					Attending an all night affair	29^k^	Social
					Night work	9^l^	Social
					Heat	4	Discomfort
[28]	Pregnant women	52^p^	LLIN	No use previous night	Experiencing labour pains	22	Other
					Heat	14	Discomfort
					Difficult to breathe under net	12	Discomfort
					Saving net for use after delivery	10	Other
					No place to tie net	6	Technical
					Net was dirty	5	Availability
					Slept elsewhere	5	Social
[29]	HIV positive adults	17	LLIN	Use < 7 nights a week or not at all	Heat	8	Discomfort
					Net unavailable	6	Availability
					Perceived mosquito density	2	PMD
					Slept elsewhere	2	Social
					Net too difficult to mount	1	Technical
					Forgot	1	Other
[30]^h^	Caregivers to children < 5	27^c^	ITN	No use previous night	Heat	9	Discomfort
					Perceived mosquito density	5	PMD
					Too difficult to get up at night	2	Technical
					Blocks the breeze at night	1	Discomfort
[31]^n^	Household members	25	LLIN	No use previous night	Perceived mosquito density	5	PMD
[32]^h^	Household head, mother or adult	277^m^	LLIN	Use < 7 nights a week	Heat	100	Discomfort
					Dislike of sleeping under net	93	Discomfort
					Perceived mosquito density	36	PMD
					Slept elsewhere	24	Social
[33]^h^	Caregivers to children < 10	116	ITN	Undefined	Heat	68	Discomfort
					Perceived mosquito density	27	PMD
[34]^n^	Pregnant women	71^0^	ITN	Use during pregnancy	Misinformation	41	Other

SS = Sample size; N = Number; a. This study seemingly reports both actual (reasons why a mosquito net was not used) and anticipated (reasons why a mosquito net may not be used) reasons for mosquito net non-use; b. Data reported by caregivers; c. The reported figure differs from that in the respective study as it excludes participants who reported non-ownership as a reason for non-use; d. Reviewer derived category of the reported reasons: 'no room to hang child's net', 'house reconstruction affects net use', 'net is too small for bed or mat', 'roof is leaking, so cannot spread the net', 'cannot hang the net'; e. Reviewer derived category of the reported reasons: 'child's net used by another', 'visitor is using child's net', child's net has been taken for mending', 'child's net was washed'; f. Reviewer derived category of the reported reasons: 'child temporarily lacks caregiver', mother or caregiver is away'; g. Reviewer derived category of the reported reasons: 'difficult to spread net over mat', 'returned home to late to put up net', 'net is too hard to put up and take down'; h. Reported additional reasons for mosquito net non-use, but these were either undefined (i.e. reported as 'other') or the respective number/percentage was not provided; i. This figure was not reported in the study (no denominator was provided); rather, it was calculated by the primary author based on the reported facts that 1347 households were surveyed and that 24% of households reported at least one household member not sleeping under a net in the past week (24% of 1347 = 323). As it was also stated that 24% of the 122 reported reasons for non-mosquito net use were due to insufficient nets, 29 (24% of 122 = 29) was subtracted from 323 = 294; j. Calculated as 36% of 122; k. Calculated as 24% of 122; l. Calculated as 7% of 122; m. The reported figure differs from that in the respective study as it excludes participants who reported non-ownership as a reason for non-usage and participants who reported their mosquito net as 'lost or stolen'; n. The sum total of 'irregular users' reported in Table 2 of this study; o. Additional reasons for non-use of a mosquito net were recorded, but were not reported in the study; p. Calculated as the 18+53 participants recorded as 'no' in the 'used in pregnancy' column in figure 1.

The reported reasons for not using a mosquito net were seemingly obtained by open-ended questioning in all nine studies, although this was only overtly stated in two [20,28]. More than one response per participant was reported in three studies [20,28,29]; in the remaining studies one or less response per participant was reported, although it was not always clear whether single or multiple responses per participant were permissible (i.e. some participants may have reported multiple reasons whilst others reported none). Four of the reported surveys were conducted during a period of peak malaria transmission [30-33], three were conducted at multiple time points across seasons [20,21,28], one in a low transmission period [29] and in one study the reference period (use during pregnancy) spanned multiple seasons, although the timing of the survey was unclear [34]. Mosquito-net non-use was independently corroborated in two studies [20,31] and was based on self-report in the others. With respect to geographic location, one study was conducted in the Solomon Islands [33], one in India [32], whilst the remaining studies were conducted in sub-Saharan Africa.

As can be seen from Table 1, six of the nine studies presented reason for not using a mosquito net data obtained from (or on behalf of) a specific sub-population [20,28-30,33,34]. Two studies presented data obtained from (or on behalf of) all household members [21,31], and one presented data obtained from adult household members only [32]. Sample sizes ranged from 17 to 294 across the nine studies, although were particularly low (< 30) in three cases [29-31]. Five studies provided a measure of mosquito net non-use that was inclusive of a time period; namely, non-use the night [28,30,31] or week [21] immediately prior to the respective survey or during the course of pregnancy [34]. In the remaining studies the non-use measure was defined (use of mosquito net < seven nights a week), but not period specific [29,32] or was not defined at all [20,33]. Five of the studies presented data pertaining to ITN that required regular insecticide re-treatment [20,21,30,33,34]; long lasting insecticide treated mosquito nets (LLIN) were the norm in the four other studies.

The predominant reasons for not using a mosquito net varied across studies, as did the number and range. Nevertheless, discomfort (primarily heat) was the predominant reason for non-use in four out of the nine studies and discomfort and perceived mosquito density were cited as reasons for non-use (although not necessarily the predominant reason) in seven out of nine and six out of nine studies, respectively. Pooling the data from across the nine studies, 948 participants reported 697 reasons for not using a mosquito net. Of these 697 reported reasons, 47.5% (331/697) pertained to discomfort, 20.1% (140/697) to social factors, 12.3% (86/697) to perceived mosquito density, 5.2% (36/697) to technical factors, 2.9% (20/697) to mosquito net availability, and 12.1% (84/697) to various 'other' factors three quarters of which specifically related to misinformation (being told not to use a provided mosquito net until after the child was born) or the onset of child birth ('experiencing labour pains'). Discomfort was the most common reason for non-use cited, irrespective of whether the net type was ITN or LLIN.

Findings from the eight 'non-specific' studies, whilst difficult to quantify or generalize, were largely consistent with those described above. Perceived mosquito density and heat (discomfort) were identified as the primary reasons for not using a mosquito net the night prior to the survey among an indeterminate number of pregnant women by Njoroge *et al *[25]. Similarly, Binka and Adongo [24] reported perceived mosquito density and heat as the reasons why 80% of 875 respondents did not use mosquito nets during the dry season (as compared to 0.3% during the rainy season). Klein *et al *[27] reported that one-third of the surveyed respondents (female household heads) from mosquito net owning homes (73% of 260) "indicated that they did not use them regularly because of the heat" (p. 385). Perceived mosquito density and low disease incidence were the reported reasons for not using a net in the 43 households in which a net was owned, but not used, in Hlongwana *et al *[22]. In Thwing *et al *[13], 68.1% of an indeterminate number of the nets reported as not hanging during the dry season "were not hanging because the respondents believed there were no mosquitoes" (p. 831). This figure fell to 6.1% during the wet season. Thwing *et al *[13] also report that, during both the wet and dry seasons, < 5% of nets not hanging were reportedly unused because of an inability to hang them. Heat along with other discomfort factors of smell and constraint were reported as the reasons why an indeterminate proportion of 471 survey respondents did not use their mosquito nets 'regularly' by Agyepong & Manderson [26].

Heat was identified as a factor contributing to partial mosquito net use (use for part of the night, but not all) by Frey *et al *[23]. In this study, the mothers of 21 children identified as having only slept under a mosquito net for part of the night prior to the survey, reported sleeping the first part of the night (with their child) outside of the house (and away from the mounted mosquito net) due to high inside temperatures. Prolonged household activity in which the child was carried on the back of his or her mother was also identified as another reason for partial mosquito net use by children in the Frey *et al *[23] study. The only 'non-specific' study in which heat or perceived mosquito density were not reported as reasons for not using a mosquito net was the Das *et al *[19] study of mosquito net texture preference. In this study, in which 60 household members were given two types of mosquito nets to trial over a 14 day period, reported reasons for not using the nets included leaking of rainwater from roof (technical), no material to tie the net (technical), use of the mosquito net by someone else (availability), or spending the night elsewhere (social).

### Qualitative data

Multiple studies were identified in which reasons for mosquito net non-use, obtained via qualitative enquiry, were presented as part of a larger body of findings. However, participants in most of these studies were not selected on the basis of being identified mosquito net owners and it was unclear, therefore, as to whether the reported reasons for non-use were based on personal experience or conjecture. Accordingly, studies in which it was not possible to distinguish the responses of mosquito net owners from non-owners were excluded from review. A total of four studies were identified in which data pertaining to mosquito net non-use, obtained via in-depth interview or focus group discussion (FGD) with mosquito net owners, were presented [35-38]. The relevant data in all four of these studies were a minor component of the reported findings and the identified mosquito net owning participants were a (identifiable) sub-sample of the respective participant groups. Nevertheless, the findings remain of interest given the review topic and are summarized below.

Alaii *et al *[35] interviewed 12 mothers from homes that had purchased their own mosquito net as part of a pre-intervention study in Western Kenya. Participants reported using the mosquito nets in the cold/rainy season and then stopping at perceived times of low mosquito density. Heat (discomfort) was most often cited as a 'problem' with mosquito nets as were 'technical' issues associated with washing or deploying the net, the size of the net (too small), and nets 'trapping' small children left unattended in bed. Atkinson *et al *[36] examined the acceptability and participant preference of three types of mosquito net via 12 FGDs in the Solomon Islands. Intermittent mosquito net use was described by 'most' participants from areas of low or seasonal mosquito nuisance. Amongst these participants mosquito net use was reportedly highest during times of perceived high mosquito density, the cooler months of the year or when a family member was sick with malaria. Howard *et al *[37] conducted six FGDs and 14 in-depth interviews with male (5 & 8, respectively) or female (1 & 6, respectively) ITN-owners living in eastern Afghanistan under the Taliban regime. Consistent with Alaii *et al *[35] and Atkinson *et al *[36], many participants reported only using the ITNs in summer when perceived mosquito densities were highest, even though a number were aware that malaria could be transmitted in other seasons. Other reasons for non-use, not related to availability, were not reported. Finally, Toe *et al *[38] interviewed 50 mosquito net users and 50 non-users as part of an LLIN acceptability study in Burkina Faso, Africa. Participants reported that they did not use their nets when they were not bothered by mosquitoes, even during periods of high malaria transmission. Low motivation for mosquito net use was evident with participants using damaged nets that could easily be repaired or replaced or reportedly forgetting to use mosquito nets. On the basis of the participant response, Toe *et al *[38] concluded that the mosquito net was primarily used to combat mosquito nuisance when necessary, rather than as a form of malaria control.

A further study was identified in which the reasons why an individual mosquito net (as opposed to mosquito net owner) was not used were examined by a mixed methodology. This study presented findings from an investigation of factors associated with the use and non-use of mosquito nets in two Ethiopian states [16]. The qualitative component of this study was integrated into a structured survey instrument and involved the use of open-ended questioning. Specifically, when a household included in the survey sample "...had an ITN that had not been slept under the prior night, the interviewer asked why, and was allowed to probe for clarification and ask follow-up questions" [16]. Resulting responses were obtained from the male or female household head and were hand recorded. Analysis of the qualitative data was not described in detail, although appeared limited to a summary of open-ended responses by an unreported method. The study took place during a period of high malaria transmission.

The survey identified a total of 1,405 ITNs owned across 857 households. Of the ITNs owned, 65% were reportedly used the night prior to the survey leaving a total of 492 unused nets. Thus, open-ended question data were presumably obtained on the reasons why these 492 nets were unused (sample size was not reported in the respective study). Seven primary reasons for mosquito net non-use emerged from the analysis of these data. Three of these reasons were consistent with the findings from the survey data reviewed above; namely, the perception that malaria or mosquitoes were not a serious problem (risk perception), the difficulty of hanging ITNs in traditional houses (technical), and the saving of ITNs for future use (other).

The four remaining themes to emerge were less evident in the survey data reviewed above, possibly reflective of the focus on whether an identified mosquito net had been used as opposed to whether an individual had used a mosquito net or not. These themes included the net being used for a purpose other than that for which it was intended, e.g. as a table cloth or room divider, the net being unused due to its poor condition, and misinformation or lack of information, especially with respect to whether the ITNs could be washed or whether they required retreatment (although, arguably, this reflects less a reason for not using a mosquito net and more confusion as to how the mosquito net should be cared for). The final theme, and the single most widely reported reason for not using a mosquito net in this study, was a perceived loss in ITN effectiveness based on the observation that dead insects no longer gathered around the net or on the belief that the ITN needed retreatment. This latter theme along with reports that mosquito nets are not being used due to their poor condition suggest that many households may be maintaining mosquito nets considered past their 'used by' date. This finding may, therefore, highlight a need to distinguish between the ownership of 'active' versus 'expired' mosquito nets in studies of this type.

## Discussion and Conclusion

Discomfort, primarily due to heat, was the most widely identified reason why mosquito net owners chose not to use a mosquito net on one or more nights in the 17 survey-based studies included in this review. The next most widely reported reason for not using a mosquito net in the survey-based studies was perceived low mosquito density; although this only accounted for 12.3% of all responses in the pooled data set (compared to 47.5% for discomfort) suggesting it was widely reported, but often at a relatively low frequency. Heat and perceived mosquito density were also consistently identified in the small number of studies presenting qualitative data, although perceived mosquito density more clearly emerged as the dominant reason for not using a mosquito net in these studies. In one qualitative study participants reported that the primary function of mosquito nets was to combat perceived mosquito nuisance (i.e. disruption of sleep) rather than malaria transmission [38]. This finding has been reported elsewhere [24,27] and indicates that the practical function of mosquito nets may differ from the intended function in some instances. The reported use of mosquito nets for fishing would be an extreme example of a problematic discrepancy between intended and practical mosquito net function [39]. Utilizing a mosquito net to minimize sleep disturbance rather than malaria transmission is considerably less problematic as protection against malaria transmission is still conferred when the net is in use. Indeed, social marketing campaigns have even promoted ITNs as a means to minimize sleep disturbance [40]. However, if this discrepancy results in seasonal or irregular use (as the evidence presented in this review indicates) then the benefit of mosquito net utilization may not be fully realized. If a primary motivation to use a mosquito net is perceived mosquito density, then it also stands to reason that in areas where mosquito density falls as a result of increased ITN coverage the continued motivation to use an ITN may decrease. In other words, the very effectiveness of the ITN may render further ITN use undesirable. If such a scenario were to eventuate, then any programmatic gains in terms of reduced malaria-related morbidity and mortality, as well as the possibility of future malaria elimination, could be potentially threatened. The relationship between mosquito net use and mosquito density may, therefore, warrant careful and ongoing investigation in areas experiencing an increase in ITN coverage.

If personal discomfort and, to a lesser extent, perceived mosquito density are the primary reasons for not using a mosquito net amongst mosquito net owners, then greater mosquito net use could potentially be achieved amongst this population via education or behaviour change communication (BCC) strategies as previously suggested [11,18]. With respect to personal discomfort, however, education or BCC strategies would do nothing to change the physical properties of the mosquito net that cause the discomfort in the first place. Thus, modifications to mosquito nets or the mosquito net using environment that render the mosquito net more comfortable would usefully complement any educational or BCC campaign.

This review identified other reasons for not using a mosquito net that may also be better addressed via strategies other than (or in addition to) education or BCC. The pooled survey data indicated that social factors, such as sleeping elsewhere, or not sleeping at all, frequently result in mosquito net non-use. Technical factors related to mosquito net use (i.e. not being able to hang a mosquito net or finding it inconvenient to hang) and the temporary unavailability of a mosquito net (primarily due to someone else using it) were also reported in the survey and qualitative studies. Social obstacles to mosquito net use may be addressed by complementary mosquito control strategies. For example, if someone is active (i.e. not in bed) during night time hours then insect repellents could be made available and their use promoted; a malaria control strategy previously trialled with some success [41,42]. Alternatively, if an individual is required to sleep somewhere other than their normal residence then additional 'travel' mosquito nets could be made available.

Additional mosquito nets in the house would usefully address the issue of non-use due to the temporary unavailability of a normally available net. Individuals who spend part of the night sleeping outdoors and part of the night sleeping indoors, a reason for 'partial' non-use identified in this review [23] and reported elsewhere [43], may also benefit from additional mosquito nets if they were able to hang them in their various sleeping areas. Nevertheless, hanging mosquito nets outdoors may continue to be problematic given current mosquito net designs and their reliance on external supporting structures. Thus, the development of 'outdoor' or 'stand alone' mosquito nets that require no external supports yet remain portable and user friendly would be beneficial. Increasing mosquito net use via a reduction in the technical difficulties associated with hanging or using a net may also best be achieved at the manufacturer level. Innovative design solutions could potentially resolve the reported difficulties in hanging a net in certain household structures (e.g. via the incorporation of internal or complementary supporting structures, compact sizes, or alternative 'clamping/tying' systems) or increase the ease with which a net may be utilized once hung. Where technical issues remain problematic consideration could even be given to the promotion of LLIN hammocks, blankets or curtains or even insecticide treated plastic sheeting (as a wall covering).

Perhaps the most important finding of this review pertains to the current state of the published research literature, which was limited at best. A basic descriptive analysis of the reported 'reasons for not using a mosquito net' data was not provided in eight out of the seventeen survey-based studies included in this review. This omission rendered it impossible to reliably interpret the relative importance of the reported findings in the respective studies. In the nine studies in which detailed descriptive analysis was presented, reliable interpretation of the data was often undermined by inadequate description of the study design. Examples included the frequent failure to report how data were obtained (e.g. in response to a structured checklist or open-ended question) or how many responses per participant were permissible. The omissions described above most probably reflect the fact that the focus of this review (reasons for not using a mosquito net as reported by mosquito net owners) was rarely a primary focus of the studies identified by the search methodology. Rather, the reviewed data were typically a relatively minor component of broader investigations of mosquito net use or malaria-related beliefs and practices. This in itself is a significant finding as it indicates an important area of investigation in the current environment of mass mosquito net distribution - why people who own/have available mosquito nets choose not to use them - has received minimal, dedicated, research attention.

In considering the recommendations made above it is also incumbent upon the reviewers to acknowledge that the survey-based data included in this review do not lend themselves readily to generalization and the pooled data must be interpreted with considerable caution. The respective surveys were variously conducted at different seasons or periods of high/low malaria transmission, a threat to cross-survey comparison and minimal data were obtained from non-African participants. In fact, the majority of reported data came from specific and diverse African sub-populations (e.g. pregnant women, caregivers of children under 5) and were often population specific (e.g. failure to use a mosquito net due to child-birth). Even seemingly generic reasons for not using a mosquito net, such as heat or perceived low mosquito density, may be more or less pertinent to specific populations, yet the quantity of currently available data is not sufficient to allow any such patterns to reliably emerge. Measures of mosquito net non-use were equally varied across the reviewed studies (rendering comparison difficult) and were often quite limited in scope; for example, not using a mosquito net the night prior to a survey. This measure, whilst convenient and clearly defined, does not allow a distinction to be made between individuals who never or rarely use a mosquito net, individuals who inconsistently use a mosquito net and individuals who usually use a mosquito net, but for whatever reason did not do so the night prior to survey. Reasons for not using a mosquito net, as well as interventions to encourage greater use, are likely to vary between these three categories of 'non-user'. Thus, the inability to identify the membership of these respective groupings, and their respective reasons for non-use, confounds informed and targeted intervention.

A further limitation of the reviewed reason for mosquito net non-use data was the paucity of qualitative investigation. The qualitative data included in the review were, as with the survey data, typically a minor component of the results presented in the respective studies. The one study in which the reported data were a primary research focus [16] employed a mixed methodology in which the qualitative component was highly structured, relatively minimal in scope and the level of analysis was limited and poorly described. This study was also distinct in that it examined the reasons why an identified mosquito net went unused as opposed to the reasons why an identified individual did not sleep under a mosquito net. Focusing on the net depersonalizes the line of questioning potentially resulting in honest more accurate data, gives a better sense of which mosquito net types (or states) may be more or less appealing and may allow use of an individual net to be tracked over time. However, by focusing on the net the reasons why the very individuals to whom the mosquito nets are provided do not always use them may go unreported or may be incorrectly reported (if someone other than the individual who would normally sleep under the net responds to the research questioning). The potential loss of, or inaccurate reporting of, relevant data inherent in study designs that focus on the net rather than the individual is of concern as it is these individuals that mosquito net promoting interventions or campaigns must target rather than the nets they choose not to use.

Taken together then, the omission of important information in many of the published survey findings, the constraints on generalization and the dearth of dedicated quantitative and especially qualitative investigation seriously undermine confidence in the reported findings of this review. The seemingly clear patterns evident in the reviewed data and the recommended interventions should, therefore, be considered highly tentative until such time as a greater quantity of dedicated, well designed and reported studies are available in the published literature. The current evidence-base is not sufficient in scope or quality to reliably inform mosquito net promoting interventions or campaigns targeted at individuals who own but do not (reliably) use mosquito nets.

A balanced consideration of the results and recommendations presented above also requires overt acknowledgement of the limitations in the review itself. Grey (unpublished) literature was excluded from review as were the numerous and varied studies that have examined barriers to mosquito net use from perspectives other than that of identified owner/non-users. The latter studies, in particular, often provide instructive data on barriers to mosquito net use and warrant review in their own right. It was the opinion of the authors, however, that the limited scope of this review was justified on the grounds that the published data broadly pertaining to barriers to mosquito net use is so extensive and varied that it is better suited to multiple 'subject specific' reviews rather than a single general review. Other 'barrier to mosquito net' review topics may include: factors predictive of mosquito net use in households with and without sufficient mosquito nets; factors predictive of mosquito net ownership - and the number of mosquito nets owned - following a mass distribution campaign; and focussed investigations into locally specific barriers to mosquito net use.

## Competing interests

The authors declare that they have no competing interests.

## Authors' contributions

JP conceived of the study, conducted the review and drafted the manuscript, MWH, MB, PMS and IM conceived of the study and critically revised the manuscript. All authors' read and approved the final manuscript.
